# Changes in Mechanical Properties of Polyhydroxyalkanoate with Double Silanized Cellulose Nanocrystals Using Different Organosiloxanes

**DOI:** 10.3390/nano11061542

**Published:** 2021-06-11

**Authors:** Jaemin Jo, Hyeyun Kim, So-Yeon Jeong, Chulhwan Park, Ha Soo Hwang, Bonwook Koo

**Affiliations:** 1 Green and Sustainable Materials R&D Department, Korea Institute of industrial Technology, 89 Yangdaegiro-gil, Cheonan-si 31056, Korea; jjm1234@kitech.re.kr (J.J.); syjeong@kitech.re.kr (S.-Y.J.); heliocity@kitech.re.kr (H.S.H.); 2 Department of Chemical Engineering, Kwangwoon University, 20 Kwangwoon-ro, Seoul 01897, Korea; chpark@kw.ac.kr; 3 R&D Center, OomphChem Inc., 1223-24 Cheonan-daero, Cheonan-si 31080, Korea

**Keywords:** cellulose nanocrystals, polyhydroxyalkanoate, polyhydroxybutyrate, biodegradable, silanization, bioplastic

## Abstract

Polyhydroxyalkanoate (PHA) is a biodegradable plastic with great potential for tackling plastic waste and marine pollution issues, but its commercial applications have been limited due to its poor processability. In this study, surface-modified cellulose nanocrystals were used to improve the mechanical properties of PHA composites produced via a melt-extrusion process. Double silanization was conducted to obtain hydrophobically treated CNC-based fillers, using tetraethyl orthosilicate (TEOS) and methyltrimethoxysilane (MTMS). The morphology, particle size distributions, and surface characteristics of the silanized CNCs and their compatibility with a PHA polymer matrix differed by the organosiloxane treatment and drying method. It was confirmed that the double silanized CNCs had hydrophobic surface characteristics and narrow particle size distributions, and thereby showed excellent dispersibility in a PHA matrix. Adding hydrophobically treated CNCs to form a PHA composite, the elongation at break of the PHA composites was improved up to 301%, with little reduction of Young’s modulus, compared to pure PHA. Seemingly, the double silanized CNCs added played a similar role to a nucleation agent in the PHA composite. It is expected that such high ductility can improve the mechanical properties of PHA composites, making them more suitable for commercial applications.

## 1. Introduction

Plastics have been widely used in human life due to their remarkable properties. They have durability, flexibility, corrosion resistance, and even low production costs, thus it is commonly recognized that plastics are vital materials in every aspect of human life [1]. Such remarkable properties of plastics, including their durability and toughness, have brought, not only enormous economic benefits, but also adverse effects on the environment [2]. The resistance to natural degradation of plastics has become a great challenge in plastic waste management, and the accumulation of end-life plastics and chemical release bring about unprecedented pressure on our environment; the so called the white pollution problem [3,4]. Recent studies have paid increasing attention to microplastics, which is a representative white pollution and a serious threat to ecological systems [2,5].

It has been reported that 26 billion tons of plastic waste will have been produced by 2050, and that half of this will be refuse, generating a serious waste management issue [6]. As the high environmental impact of plastic has notably been acknowledged over the past decades, regulation on plastic use, especially for single-use plastics, has been tightened globally as a front control strategy [7,8,9]. The EU decided to implement guidelines for banning ten non-degradable plastics, including straws and tableware, which can be replaced with eco-friendly materials. Such global trends have led to a drastic increase in demand for biodegradable plastics (BPs) derived from renewable resources. The development and production of BPs seem to be the least expensive and most effective way to control plastic waste in the long term.

The market for bioplastics, including BPs, was 2.1 million tons in 2020, which accounts for only 0.5% of the total plastic market. Nevertheless, growing concerns over microplastics and global warming encourage the widespread use of biodegradable plastics, which are not just fragmented to smaller sizes, but truly degradable, e.g., polylactic acid (PLA), polycaprolactone (PCL), polybutylene succinate (PBS), and polyhydroxyalkanoate (PHA) [10,11].

PHA is a natural thermoplastic polyester polymer that can be totally decomposed in soil, as well as the marine environment, which is a big advantage over PLA [12]. It can be used for various purposes, such as food-packaging materials or medical applications, due to its biocompatibility and barrier property for water and UV rays [13,14]. Nonetheless, it is still challenging to manufacture a final product composed of 100% PHA, considering the high manufacturing cost and processing instability. Thus, it is strongly desired to enhance its processability and mechanical properties, to make PHA more industrially feasible for commercial applications in the BPs market.

The thermal and mechanical properties of PHA can be modified by chemical modifications, co-polymerization, and blending with nanoparticles or additives [14,15,16,17]. Nanomaterials, such as clay, inorganic nanoparticles, carbon nanotube, plasticizer, lignin, and nanocellulose, have been widely added to produce PHA-based nanocomposites with the desired properties [10,13,14,15,16,17,18,19,20]. Blending with renewable and biodegradable materials is advantageous for producing composites with enhanced properties, without compromising their degradability.

Cellulose is considered a sustainable alternative source to petroleum-based synthetic products, and nanocellulose has received special attention as a strong reinforcement component in bio-composites, due to its enhanced dispersibility compared to microfibers and the tunability of its surface chemistry. There are several kinds of nanocellulose, and cellulose nanocrystals (CNCs) are the most common form of cellulose-based nanofiller. CNCs are mainly produced by acid treatments, and the diameter and length of CNCs are 5 to 20 nm and 100 nm to several μm, respectively [21,22]. Its inherent structural properties, such as its significant aspect ratio, and high strength, stiffness, and surface area, make CNC favorable as a nanofiller in composites.

However, CNCs have abundant OH groups giving high hydrophilicity and low compatibility with a hydrophobic plastic matrix. Thus, surface modification of CNCs must be conducted to improve the compatibility with hydrophobic PHA. In order to solve the issue, a variety of surface functionalization strategies have been applied to CNCs [10,14,18,20,23,24,25,26,27,28,29,30]. For example, the methylation process decreases the water absorption tendency of CNCs through hydrophilic OH group substitution with hydrophobic methyl, ethyl, or acetyl groups [18,26]. The addition of surfactants and grafting may be promising routes for the hydrophobization of CNCs [10,14,23,24,25,27,28,29,30].

Silanization is an effective grafting method for obtaining hydrophobically modified nanocellulose, by substituting OH groups on the surface with various Si-containing end groups [14,23,24,30]. Many studies used tetraethyl orthosilicate (TEOS) and methyltrimethoxysilane (MTMS) as organic precursors of alkoxysilane for the surface modification of CNCs, due to their low cost, low toxicity, and chemical stability [31,32]. It was reported that silanized cellulose nanofibers showed good adhesion with PHA based composites and contributed to the enhancement of their mechanical properties [23,33]. For example, Frank et al. (2018) reported that silanized cellulose nanofibers treated with a hydrophobic alkyl chain containing silanes, e.g., MTMS or propyl trimethoxysilane, showed improved compatibility with a PHA polymer matrix, compared to neat cellulose nanofibers. A similar approach with CNCs could offer a route to prepare renewable material-based fillers suitable for composite productions with a hydrophobic polymer matrix.

This study aimed to enhance the mechanical properties of PHAs through melt-extrusion blending with silanized CNCs. The silanized CNCs were prepared via surface modification of CNCs using organosilanes with different end groups. Here, the silanization of CNCs was conducted using TEOS or MTMS. It is well-known that the surface modification of cellulose using TEOS can increase the interaction between the composite phases [33,34]. MTMS is an alkoxysilane containing CH_3_ groups, which is widely used for increasing the hydrophobicity of nanocellulose or other OH group-rich surfaces [23,24]. Thus, single or multiple steps of silanization were performed to obtain a CNCs-based filler with dimensional stability and hydrophobicity. Their dispersibility in the PHA composites varied with their surface characteristics and drying methods, and their effects on the mechanical properties of the final product PHA composites were determined.

## 2. Materials and Methods

### 2.1. Materials

For PHA, poly-3-hydroxybutryate (P3HB) and poly-4-hydroxybutryate (P4HB) mixture were kindly provided by CJ CheilJedang (Seoul, Korea). CNCs were purchased from CelluForce Inc. (Montreal, QC, Canada). Hydrochloric acid (HCl, 1 N) and MTMS (96%) were purchased from Daejung Chemicals & Metals (Siheung-si, Korea). Ammonia solution (25.0–30.0%), isopropyl alcohol (IPA, >99.9%), and ethyl alcohol (EtOH, 94.5%) were purchased from Samchun Chemicals (Seoul, Korea). TEOS (98%) was purchased from Sigma-Aldrich (St. Louis, MO, USA).

### 2.2. Surface Modification of CNCs

**Figure 1:**
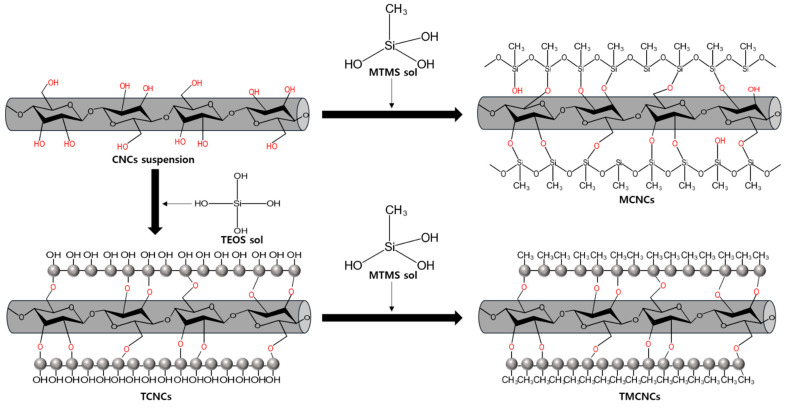
Schematic illustration of surface treatment of CNCs using TEOS and MTMS.

Surface treatments of CNCs using MTMS and TEOS were conducted in a continuous sol-gel process at ambient conditions, as described in Figure 1. When adding MTMS into a weak acidic aqueous solution, it is pre-hydrolyzed and condenses with the OH group-rich surface of CNCs. As water is removed via the drying process, the silanized CNCs with CH_3_ end groups are formed.

First, CNCs treated only with MTMS was prepared, following a previous study [24,35]. MTMS was dropwise added into a weak acidic aqueous solution (pH ≈ 4) at 2 wt% concentration and hydrolyzed. The MTMS solution was added dropwise to an aqueous suspension at pH 4 containing CNCs at 1 wt% of solid contents, and the mixture was stirred for 2 h at 500 rpm. At the end of reaction, the mixture was centrifuged at 1000 rcf (relative centrifugal force) for 5 min, and washed three times with EtOH. Finally, the obtained hydrophobically treated CNCs using MTMS (MCNCs) were freeze-dried and then dried at 150 °C in a vacuum oven for heat curing [24].

**Figure 2:**
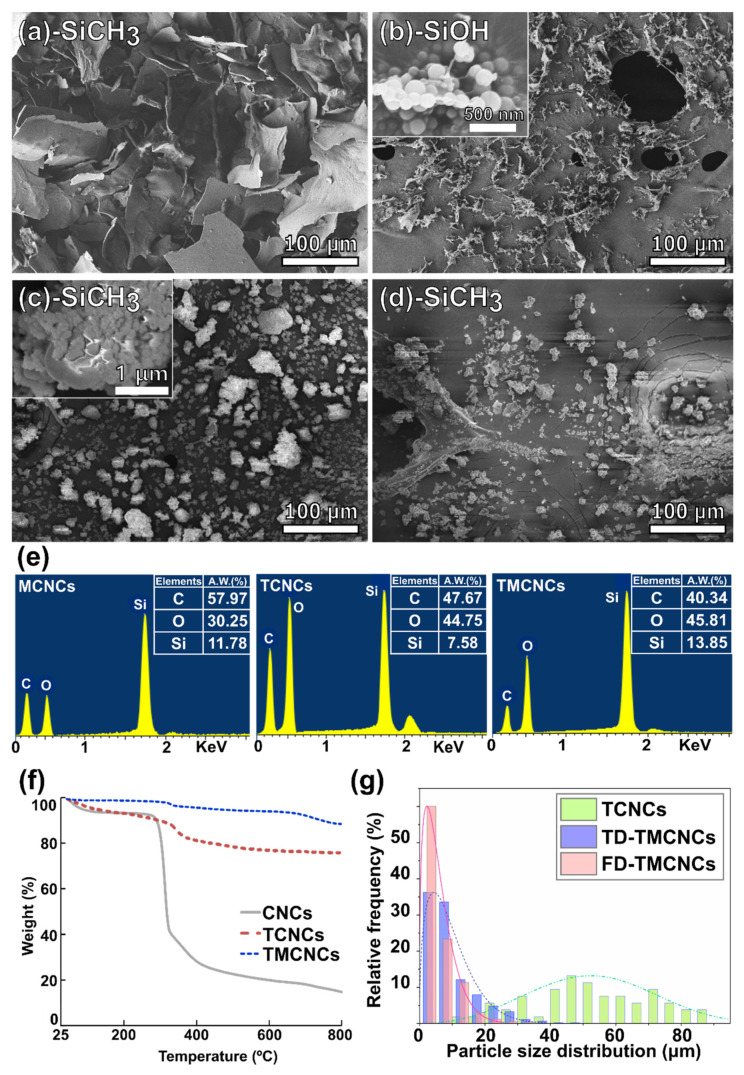
FE-SEM images of (**a**) MCNCs, (**b**) TCNCs, (**c**) TD-TMCNCs, (**d**) FD-TMCNCs, with their terminal functional group written in the panels, (**e**) elemental analysis by EDX, (**f**) TGA, and (**g**) histogram of particle size distribution including average length of TCNCs and particle diameters of TD- and FD-TMCNCs.

TEOS core-shell coating of CNCs was implemented following a modified Stöber method, as shown in Figure 1 [36]. For TEOS sol-gel coating, CNCs were suspended in a mixture of alcohol and water. The addition of TEOS allowed their hydrolysis and initiated their condensation in situ onto the CNCs in an alkaline condition. Meanwhile, nucleation of the silica precursor resulted in spherical growth of SiO_2_ on the surface of CNCs, as illustrated in Figure 1 and presented in Figure 2b,c. According to the literature [36], IPA is a favorable solvent, over ethanol and methanol, forming an even distribution of SiO_2_ on the surface of nanocellulose. CNCs suspension of 1 wt% solid content (30 mL) was added to a beaker containing 120 mL of 82% IPA, then 2.8 mL of ammonia solution was added. TEOS of 3 mL was added dropwise and the mixture was stirred for 12 h at 500 rpm. The reaction mixture was centrifuged at 1000 rcf for 5 min, and washed three times with EtOH. The CNCs treated only with TEOS are denoted as TCNCs. The TCNCs were thermally dried at 105 °C.

As illustrated in Figure 1, MTMS can condense with a SiO_2_-rich surface, shielding its terminal OH groups from CH_3_ (so called CH_3_ end capping reaction) and obtaining a hydrophobic surface. For the hydrophobic coating of TCNCs, 3% (*v/v*) of MTMS was added dropwise into a coating medium of TCNCs prepared by the same method as above, without centrifugation. The reaction was stirred for 8 h at 500 rpm. The CNCs with CH_3_ ends treated by TEOS and MTMS in series were named TMCNCs. TMCNCs thermally dried in an oven at 105 °C overnight were denoted as TD-TMCNCs and freeze-dried counterparts were denoted as FD-TMCNCs. TMCNC samples dried by different methods underwent thermal heat-curing at 150 °C in a vacuum oven, in the same manner as the TCNCs.

### 2.3. Compounding of PHA/CNCs Composites

PHA composites blended with 10 wt% of untreated-CNCs, MCNCs, TCNCs, TD-, and FD-TMCNCs, and 5 wt% of FD-TMCNCs were prepared by hot melt-extrusion using a Minimax Molder (Custom Scientific Instruments, Easton, PA, USA). The crucible temperature was maintained at 165 °C, and the sample mixtures were melted for 1 min followed by blending at 50 rpm for 5 min. Each composite was pressed to be a plate at 165 °C under a pressure of 5 MPa for 2 min, using a hydraulic hot press machine (QMESYS, Uiwang-si, Korea) and was cut into a tensile test specimen following the JISK 6251 standard. The composite mixed with (x% of) a silanized CNCs was denoted as “PHA/(x%-) name of the CNCs”, e.g., PHA/TCNCs, PHA/5% FD-TMCNCs, and PHA/10% TD-TMCNCs.

### 2.4. Characterizations of CNCs and PHA/CNCs Composites

The surface modification of CNCs using MTMS and TEOS was confirmed by Nicolet 6700 Fourier transform infrared (FT-IR) spectrometer (Thermo Scientific, Waltham, MA, USA). KBr pellets were prepared and milled together with untreated CNCs and each hydrophobic coated CNC (MCNCs, TCNCs, TMCNCs) in agate mortar. The FT-IR spectra were acquired under transmission mode with 32 scans from 500 to 4000 cm^−1^ at the spectral resolution of 8 cm^−1^.

Hydrophobicity of the CNCs samples was evaluated by water contact angle (WCA) measurements using the sessile droplet technique with smart drop (FEMTOBIOMED, Seongnam-si, Korea). WCA was measured when 8 uL water droplet fell on the top of each sample placed on a slide glass. The baseline was set at the interface between the water droplet and the sample. Each chemically modified CNC (dry weight 0.1 g) was suspended in 20 mL of water and chloroform, to examine the dispersibility of the CNCs depending on their terminal end groups.

A tensile test was performed using a Universal Testing Machine (UTM, QMESYS, Uiwang-si, Korea), equipped with a 5 kgf load cell at an ambient condition, following the JISK 6251 standard method. Specimens of 10 mm width, 30 mm length, and 1 mm thickness were tested at a strain rate of 30 mm/min and the fractured area was observed by electron microscopy. Significance of the differences of triplicate measurements of tensile stress at break, Young’s modulus, and elongation at break were statistically analyzed using one-way analysis of variance (ANOVA) using SPSS (IBM, Armonk, NY, USA) software.

The different surface morphologies of the CNCs and the fractured composites after tensile test were observed by field emission scanning electron microscopy (FE-SEM, JEOL, Akishima, Tokyo, Japan) operated at a 5 kV accelerating voltage. Specimens were sputtered with platinum to prevent surface charging effects. Energy-dispersive X-ray spectroscopy (EDX, INCA Penta FET-X3, Oxford Instruments, Abingdon, UK) was operated at a 15 kV accelerating voltage to investigate the dispersion of the CNCs in the PHA composites. Particle size distributions of the silanized CNCs were obtained from image analysis of microscopic images using the ImageJ software 1.53e [37]. The gray scale SEM images were binarized followed by thresholding above the noise level and using the particle analysis routines in ImageJ, assuming elliptical pores.

Melting and crystallization behaviors of the pristine PHA and the PHA/5% FD-TMCNCs composites were analyzed by differential scanning calorimetry (DSC, PerkinElmer, Waltham, MA, USA). Samples of ≈6 mg were heated from 30 °C to 200 °C at 20 °C/min and kept for 5 min to erase thermal history. Erased thermal history samples were cooled from 200 °C to −50 °C at 10 °C/min. Then, they were heated again from −50 °C to 200 °C at 10 °C/min for determination of glass transition temperature (T_g_), crystalline temperature (T_c_), cold crystallization temperature (T_c*_), and melting temperature (T_m_).

## 3. Results

### 3.1. Morphological Analysis of CNCs

Figure 2a–d shows microscopic images of the CNC samples prepared by different organosiloxane treatments and drying methods. To confirm the silanization of the CNCs, a complementary analysis using EDX and TGA was implemented (Figure 2e,g). The particle size distributions of the CNCs treated with different silanization strategies were analyzed based on the microscopic images above, and the histogram is displayed in Figure 2g.

MTMS only treated CNCs (MCNCs) formed a bundle of sheet-like particles larger than 150 μm diameter (Figure 2a). Due to the crosslink between MTMS silanol and CNCs, the CNCs were agglomerated, not present as individual particles. Thus, it was difficult to estimate the exact average particle size. TEOS-treated CNCs had a relatively lower average particle size than the MCNCs, and they are compared in Figure 2g. Cellulose is mineralized via TEOS treatment, which can decrease the interaction with water and between fibers, consequently, preventing the aggregation of CNCs to larger particles [34,38]. Therefore, TCNCs, with fibrous core-shell structures, few-micrometer-widths, and 60 μm average length scales were obtained (Figure 2b). A magnified view in Figure 2b presents spherical nanoparticles on the surface of the TCNCs, which are derived from nucleation of TEO during the surface coating of CNCs.

The same morphology was observed in the TMCNCs, as shown in Figure 2c. Spherical SiO_2_ nanoparticles resulting from TEOS treatment also appeared on the surface of the TMCNCs, but a thin coating of MTMS silanol and changed surface characteristics needed to be confirmed by other analytic methods, such as FT-IR and WCA. Raabe et al. (2014) reported that the high surface area with presence of abundant spherical nanoparticles after TEOS treatment is likely to greatly improve their interaction with the CNCs and polymer matrix, if they have good adhesion, and consequently the mechanical properties as well [34]. After the CH_3_ end capping reaction using MTMS, TCNCs agglomerated, forming smaller granular particles (Figure 2c), which seemingly can be attributed the hydrophobic effect in the aqueous silanization process. This implies that the surface characteristics of the TMCNCs changed from hydrophilic to hydrophobic (Figure 2c,d).

The size distribution of the particles varied depending on the drying method. The average diameter of the FD-TMCNCs was measured as 10.67 μm. More than 60% of the FD-TMCNCs particles had a less than 5 μm diameter, whereas less than 3% of particles had a diameter larger than 20 μm. The average diameter of TD-TMCNCs was 14.15 μm, which was larger than the former by 32.6%. TD-TMCNCs particles had more than 10% of particles larger than 20 μm in diameter, and this meant that the thermal drying after MTMS treatment of TCNCs led to particle agglomeration. Comparing the particle sizes of the silanized CNCs (MCNCs > TCNCs > TMCNCs), double silanization, which is a serial polysiloxane treatment using TEOS and MTMS, was advantageous for obtaining CNC-based fillers with smaller average diameter. FD-TMCNCs had a narrower particle size distribution compared to TD-TMCNCs. It has been reported that SiO_2_ nanoparticles with narrow size particle distribution have a better dispersibility in a polymer matrix [39].

The EDX spectra (Figure 2e) display the presence of Si element of each CNC, which means that the silanization was successfully carried out. In particular, the TMCNCs had a higher Si content than the TCNCs, and the same trend was observed in TGA. TGA was carried out to quantitatively analyze Si contents in the CNCs and the results showed that TCNCs contained ≈60% of SiO_2_ and the TMCNCs ≈72%. The increased silica content of the TMCNCs originates from the condensation of MTMS. The weight loss of CNCs and TCNCs at the temperature range lower than 200 °C can be considered as removal of water, whereas a smaller drop was found in TMCNCs. This is because of the significant reduction of OH groups and hydrogen bonding with moisture at the surface of the TMCNCs as their terminal groups change to CH_3_.

Likewise, it is expected that the drying method and surface treatment method, by determining the chemical and physical properties of silanized CNCs, are important factors for their compatibility with PHAs and the mechanical behaviors of the composites. The surface characteristics of the silanized CNCs and their dispersion behaviors in a PHA polymer matrix are investigated in the following section.

### 3.2. FT-IR Spectra of the Silanized CNCs

**Figure 3:**
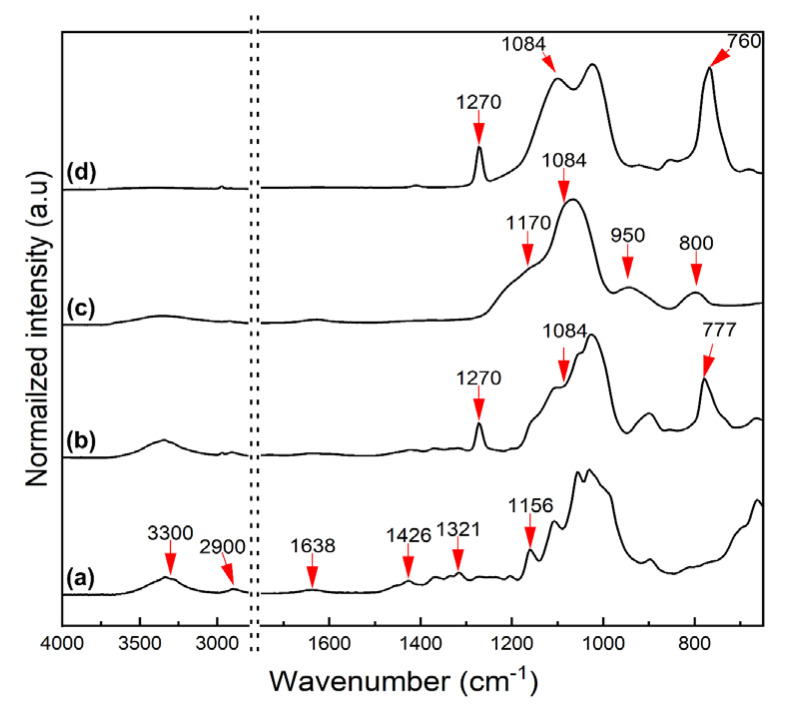
FT-IR spectra of (**a**) CNCs, (**b**) MCNCs, (**c**)TCNCs, and (**d**) TMCNCs.

Silanization of CNCs using polysiloxanes was confirmed by FT-IR, following the literature [32,40,41], and as shown in Figure 3. In the spectra in Figure 3a, the peaks derived from the surface groups of CNCs were observed at 1056 cm^−1^ (C-O-C stretching), 1321 cm^−1^ (C-H deformation), 1426 cm^−1^ (-OCH bending), 1638 cm^−1^ (H-O-H bending), and 3300 and 2900 cm^−1^ (O-H stretching). The FT-IR spectra of MCNCs (Figure 3b) show the peaks at 777 and 1270 cm^−1^, which are assigned to Si-O bending and Si-CH_3_ vibration, respectively, and derived from the silanization of CNCs using MTMS. A slight growth of the peak at 1084 cm^−1^ (Si-O-Si stretching) also appeared. In Figure 3c, the peaks indicating the chemical structure of CNCs disappear, whereas the peaks corresponding to the silanized surface appear at 800 cm^−1^ (Si-C vibration), 950 cm^−1^ (Si-OH), 1084 and 1170 cm^−1^ (Si-O-Si stretching). Particularly, the TCNCs were fully covered by SiO_2_ nanoparticles with OH end groups, which can be confirmed by the peak at 950 cm^−1^. By coating with MTMS on the TEOS treated CNCs, the OH end groups of SiO_2_ are shielded by CH_3_ groups for simultaneous hydrophobization. Thus, the Si-CH_3_ peaks at 760 and 1270 cm^−1^ are pronounced, while the one at 950 cm^−1^ (Si-OH) is significantly decreased (Figure 3d). The FT-IR spectra demonstrated that the surface functionalization of CNCs via silane treatment was successfully completed, while the actual hydrophobization was evaluated by WCA measurements and solvent dispersion.

### 3.3. Hydrophobicity Determination of CNCs

**Figure 4:**
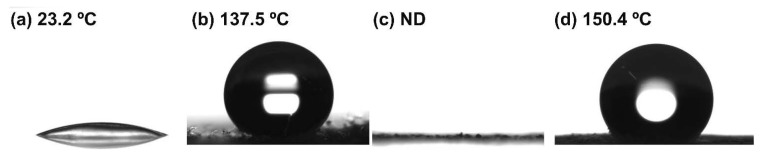
Water contact angles of (**a**) CNCs, (**b**) MCNCs, (**c**) TCNCs, and (**d**) TMCNCs (ND: not detected).

WCA is a direct indicator for determining the surface characteristics of a material as hydrophilic or hydrophobic. The WCA of each CNC film was varied depending on their surface functional groups, obtained from different organosilane treatments. A low WCA measured on the CNC films was attributed to abundant OH groups on the surface. On the contrary, a film prepared with MCNCs showed a 137.5° of contact angle (Figure 4b), which means the introduction of CH_3_ end groups made its surface characteristics hydrophobic. For the TCNCs film, the WCA could not be measured, as the water drop quickly spread through its OH-rich group and rough surface by the capillary effect. TMCNCs showed a WCA higher than 150° (Figure 4c), close to a superhydrophobic surface. This result indicated that the hydrophobization of the CNCs was successfully achieved by serial silanization using TEOS and MTMS, and hence an improvement in compatibility with PHA was expected.

**Figure 5:**
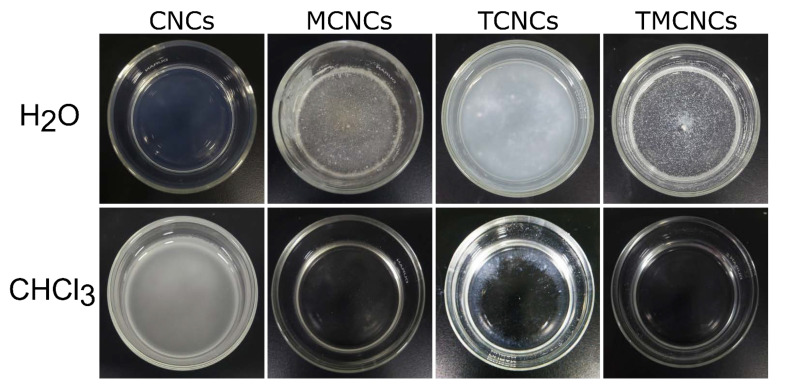
Dispersion state of CNCs and silanized CNCs in H_2_O and CHCl_3_.

As a complementary experiment, the dispersion behaviors of the CNCs in solvents with different polarities were examined. Figure 5 exhibits the dispersion states of CNCS in solvents with different polarities, H_2_O and CHCl_3_. Pristine CNCs were well-dispersed in H_2_O, but poorly mixed with CHCl_3_. TCNCs were sufficiently dispersed in H_2_O due to hydrogen bonding between H_2_O and TCNCs, but the micron sized particles made the suspension look hazy. On the contrary, they partially precipitated in CHCl_3_ (the white ring shown in Figure 5). In the aqueous suspensions containing MCNCs and TMCNCs, the particles aggregated with each other, being separated from the H_2_O because of the hydrophobic effect. The hydrophobically treated CNCs, MCNCs, and TMCNCS were well-dispersed in CHCl_3_, a solvent with low polarity. Such dispersion behavior indicates that their surface characteristics became hydrophobic via a CH_3_ end-capping reaction using MTMS. These results are consistent with a previous study, where hydrophobically treated cellulose-based materials altered their dispersion behaviors in polar and non-polar solvents [23,32].

### 3.4. Dispersibility of CNCs in the PHA Composites

**Figure 6:**
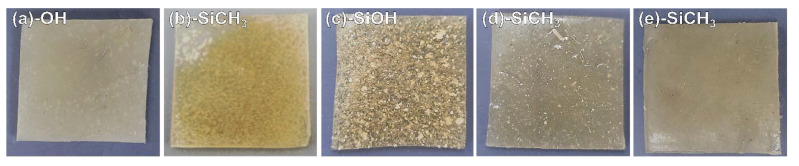
Nanocomposites containing 10 wt% of (**a**) CNCs, (**b**) MCNCs, (**c**) TCNCs, (**d**) TD-TMCNCs, and (**e**) FD-TMCNCs, the terminal functional group of each CNC is written in each panel.

The compatibility of CNCs with PHA depending on surface treatment and drying was evaluated visually, see Figure 6. The CNCs, MCNCs, and TCNCs showed poor compatibility with PHA (Figure 6a–c), with lots of grains, while seemingly the CNCs agglomerated. As expected, a clear reduction in small particles was confirmed (Figure 6d,e) in a sample of PHA/TMCNCs composite. In the case of the PHA composites with untreated CNCs and TCNCs, aggregated CNCs particles appeared, which seem attributable to the different polarity between raw CNCs and PHA (Figure 6a,c) [42]. This result was consistent with their dispersion behavior in the non-polar solvent, CHCl_3_, as shown in Figure 5. In previous studies, similar aggregations of nanocellulose in a hydrophobic polymer matrix were reported when their adhesion at their interface was poor [23,25,43]. MCNCs also show poor dispersibility in PHA (Figure 6b), despite of their hydrophobic surface characteristics. The average particle size of MCNC is assumed to be more than 1–2 orders of magnitude larger than that of TMCNCs. MCNC is produced by a crosslinking reaction between CNCs and silanol. It seems that the lower molecular mobility and flexibility of MCNCs and the large particle size negatively affect their dispersion in a PHA polymer matrix [32]. The FD-TMCNCs blended PHA composite showed a better dispersion than that of the TD-TMCNCs, with no visible agglomeration. It was considered that the small particle size of the TMCNCs, with the narrow distribution obtained by TEOS treatment and hydrophobic surface through MTMS treatment, effectively helped improve their compatibility with PHA. This is in agreement with a previous study [34].

**Figure 7:**
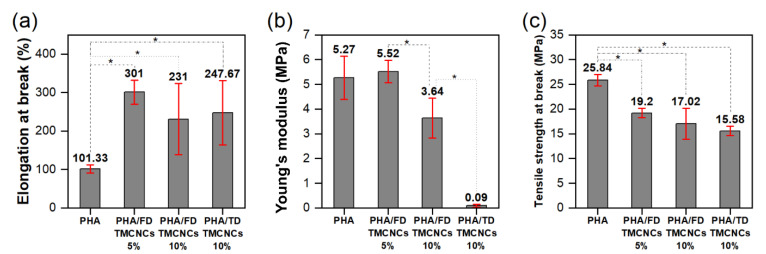
Post hoc test of mechanical properties of the PHA/TMCNCs composites, (**a**) elongation at break, (**b**) Young’s modulus, and (**c**) tensile strength at break (*–difference between two values is statistically significant at 0.05 level).

Such poor compatibility between PHA and CNCs would negatively affect the mechanical properties of the composites and jeopardize the durability of the polymer matrix, which is the main load-bearing part. Therefore, only the PHA composites containing TMCNCs were chosen for further mechanical property testing. Due to their poor mechanical properties, the PHA with untreated CNCs, MCNCs, and TCNCs were excluded from the mechanical property determination in Figure 7, but part of their fractured area is observed together in the following section.

### 3.5. Mechanical Properties of PHA/TMCNCs Composites

The mechanical properties of PHA/TMCNCs composites were analyzed by multiple comparison analysis, called a post hoc test, using a least significant difference (LSD) method, as shown in Figure 7. The post hoc analysis was conducted to compare the variance between the sample groups and within each group. The results of the multiple tests were valid when p-value of ANOVA was less than 0.05 and the F-value obtained was higher than 4.07. From the ANOVA results in Table 1, it can be verified that TMCNCs’ compounding ratio significantly affected the mechanical properties. The tensile strength of a pure PHA was in the same range as the tensile strength found (14–27 MPa) in the literature [44,45]; however, decreases in the tensile strength and elongation at break of PHA/TMCNCs composites were recorded compared with the pure PHA. Although the difference in the elongation at break and the tensile strength at break of the PHA/10% FD-TMCNCs and the PHA/10% TD-TMCNCs was negligible, the latter showed a significantly lower Young’s modulus. It is assumed that the TD-TMCNCs having a large and wide particle size distribution led to their poor dispersibility in the PHA polymer matrix and non-uniform load distribution on the composites, consequently resulting in deformation at an early stage of tensile testing, when a very low stress was applied.

The PHA/5% of FD-TMCNCs showed the highest elongation at break (301%) and the lowest decrease in tensile strength by 26% compared to that of the pure PHA. The Young’s modulus of the PHA/5% FD-TMCNCs composite was almost the same as that of the pure PHA. This means that the particles were mixed well in the PHA polymer matrix, and its elastic behavior before the yield points was almost the same as that of the pure PHA. The addition of the double-silanized CNCs into PHA was not effective for improving the tensile strength of the composites but contributed to drastically increasing the elongation at break. It is expected that such an enhanced ductility can improve the processability in manufacturing products in future applications, e.g., food packaging or films [42].

Similar results were found in a previous study on PHA blended with various plasticizers and nucleating agents [45,46,47]. El-Hadi et al. (2002) explained that the decrease of tensile strength and increase in elongation at break was related to the size distribution of spherulites in the PHA [45]. Generally, it is known that the poor mechanical properties of PHA is attributed to its low crystallinity, slow crystallization, and broad size distribution of spherulites. The presence of large spherulites causes brittleness of the PHA with a low strain. Adding nucleation agents can promote the growth of small crystals during the crystallization process and reduce the number of large spherulites [45]. Here, it might be considered that the 5% of FD-TMCNCS added had a similar role to a nucleation agent, improving the crystallinity properties through a narrow, homogeneous size distribution, and excellent dispersibility, as proven by visualization. The type of nucleation agent and crystallization condition greatly affect the size of the spherulites and the mechanical properties of the PHA [13,19,25,45,48].

**Figure 8:**
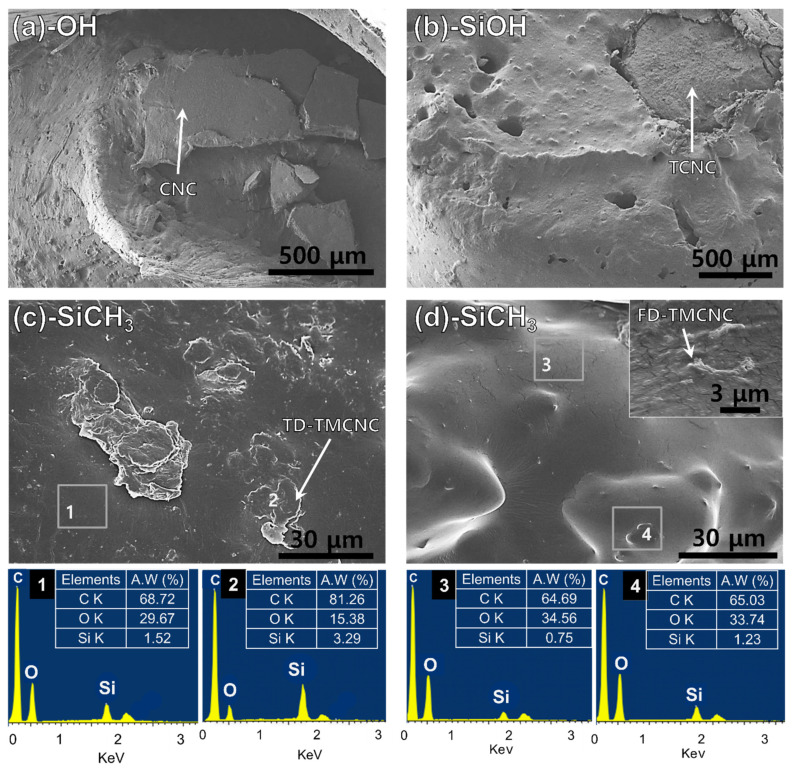
SEM images of fractured cross-sections of PHA composites with 10 wt% of (**a**) non-treated CNCs, (**b**) TCNCs, (**c**) TD-TMCNCs, (**d**) FD-TMCNCs and EDX spectra, the terminal functional group of each CNC is written in the panel.

The dispersion of each silanized CNC in the PHA matrix was evaluated by observing the fractured area of the tensile test specimens. In Figure 8a,b, particles with diameters larger than 500 μm are observed in microscopic images, which look distinctively disparate from the PHA. The particle shown in Figure 8a looks dimensionally much larger than the non-treated CNC. The TCNC particles observed in Figure 8b are also much larger than those in Figure 2b. It seems that the CNCs with OH end groups likely aggregate with each other rather than breaking into smaller pieces, which is consistent with their visual observation by naked eye in Figure 5. Figure 8b more evidently displays voids in the PHAs where TCNCs were pulled out, being subjected to tensile forces and the separation at the interface between the TCNCS and PHA matrix. This is seemingly caused by the different polarity of the PHA and the CNCs based materials with an OH group abundant hydrophilic surface and their low interfacial adhesion [33,49]. Consequently, such an inhomogeneous distribution results in the poor mechanical strength of the PHA/CNCs and PHA/TCNCs composites.

On the contrary, TMCNCs were well-mounted onto the PHA polymer matrix, with no voids or separations observed at their interfaces. This implies that introducing CH_3_ groups on the surface of TCNCs using MTMS successfully improved the affinity between PHA and TMCNCs, as observed in Figure 8c,d; which is in the same range as those in Figure 2c,d, respectively. The FD-TMCNCs/PHA composite had a smoother surface, with less defects, compared to the others shown in Figure 8a–c. Regarding the particle size of FD-TMCNC, the majority of them were a few micrometers in diameter, which is observed in a highly magnified view (top right side of Figure 8d).

The EDX spectra show the Si composition at different points in the PHA composites with TMCNCs. The points 1–4 were chosen as the representative parts, and 1 and 3 seem more likely to contain PHA, while 2 and 4 have more TMCNCs. Even though the TMCNCs are not visible at points 1 and 3, they also contain some amount of Si, which means that the TMCNCs are well-dispersed all over the PHA composites. At points 2 and 4, a relatively higher amount of Si was detected compared to the others, as expected. The difference in Si composition between point 1 and 2 in TD-TMCNCs was larger than that between point 3 and 4 in FD-TMCNCs. This means that FD-TMCNCs were better distributed in the composites due to better surface adhesion with PHA and less aggregation. The visualized composite samples in Figure 6 have the same trends as the microscopic images, where FD-TMCNC is the most uniformly compounded with PHA, without visible unmixed particles. The results of the distribution status of the silanized CNCs support the effect of the compatibility between fillers and the polymer matrix on their mechanical properties. Therefore, it can be concluded that both the surface hydrophobicity of the filler and the particle size distribution, determined by surface treatment and drying method, significantly affect their dispersibility in the composites and the mechanical properties of the final composite products containing them.

These results demonstrate that the surface treatment and drying methods of the silanized CNCs affect the average size distribution, dispersibility in the polymer matrix, interfacial strength between them, and consequently the mechanical properties of the final product composites. It was confirmed that the double silanization strategy using different polysiloxanes (TEOS and MTMS) and the freeze-drying method effectively improved their interfacial adhesion in PHA and the ductility of the PHA composites.

### 3.6. Thermal Behaviors of the PHA/5% FD-TMCNCs Composite

**Figure 9:**
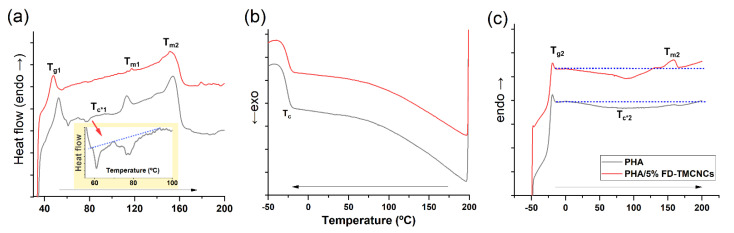
DSC thermograms of non-isothermal (**a**) 1st heating traces, (**b**) cooling traces, and (**c**) 2nd heating traces of the composites PHA (grey solid line) and PHA/5% FD-TMCNCs (red solid line), T_g1_ and T_g2_: Glass transition temperature measured in the 1st and 2nd melting process, T_c*1_ and T_c*2_: exothermic cold crystallization temperature during 1st and 2nd heating, T_c_: crystallization point, T_m1_, T_m2_, and T_m3_: melting point at different points, blue dotted line: base line to highlight cold T_c*1_ and T_c*2_).

It has been widely reported that the crystallization behaviors of a PHA can be related to its mechanical properties [45,50]. To elucidate the contributions of CNCs in changing the mechanical properties of a composite, the thermal behavior of pristine PHA and PHA composite containing 5% FD-TMCNCs was analyzed using DSC (Figure 9). Note that the thermal behavior of the composites shown in Figure 9a is associated with the different mechanical behaviors of the composites after production by melt-extrusion and storage at room temperature. Going through cooling and a second heating, the microstructure of the PHA composites changed, consequently showing different thermal behaviors in the second heating (Figure 9c). Nonetheless, the result can support the relationship between crystallization rate, the results of Figure 9a, and mechanical properties of the composites.

In the 1st heating scan curve, the pristine PHA sample had a T_g_ 52.3 °C, slightly higher than that of the PHA/5% FD-TMCNCs composite. A peak of T_m1_ (106–119 °C) and exothermic cold crystallization peaks (T_c*1_, 60–100 °C) appeared in the pristine PHA, while they were negligible in the CNCs containing counterpart. The increase of T_c_ may have been associated with the increase of crystalline regions, crack density, and spherulite sizes, and consequently the brittleness of the polymer [51]. The presence of two melting peaks (T_m1_ and T_m2_) in the pristine PHA implied the inhomogeneous growth of crystals, as T_m1_ is attributed to the melting of the primary crystals and T_m2_ to the melting of crystals from recrystallization [52]. Cold crystallization only being shown in the pristine PHA means its crystallization rate was slower than that of the PHA/5% FD-TMCNCs [53]. The absence of those peaks in the PHA/5% FD-TMCNCs composite means that the addition of FD-CNCs suppressed the inhomogeneous growth of crystals.

There was no significant difference in Tc for the two composites in the cooling scan curve (Figure 9b) and T_g2_ in the second heating scan curve (Figure 9c). Both composites showed broad exothermic peaks of cold crystallization during the second heating (below the dotted guideline in Figure 9c). The PHA-5% FD-TMCNCs composite showed an exothermic peak in the range of 0 °C to 129 °C, with a peak point at 90 °C, which was not observed in the broad peak of the PHA in the range of 0 °C to 191 °C. The melting point of the PHA/5% FD-TMCNCs composite corresponded to a single melting point T_m3_ (peak point at 158 °C), whereas no peak for T_m_ was found in the pristine PHA. The T_m_ of such an amorphous PHA is difficult to measure by DSC analysis. The presence of the peaks at T_c*2_ and T_m3_ in Figure 9c can be interpreted as crystals being formed in the PHA/5% FD-TMCNCs as the crystallization rate slightly increased with the addition of the FD-TMCNCs. It is assumed that such an increase in the crystallization rate contributes to the formation of fine spherulites in the PHA/5% FD-TMCNCs composite and the enhancement of plasticity.

It is known that slow crystallization kinetics can induce the formation of rigid amorphous regions and spherulites with large and inhomogeneous size distributions and more cracks and lamella crystalline plates [53]. Such a microstructure can lead to a decrease of ductility of amorphous pristine PHA. Zhang et al. [51] reported that a decrease in radius of spherulites results in a reduction of cracks in spherulites and an increase of elasticity of PHA. According to the results above, it is assumed that the pristine PHA composite formed by the melt-extrusion process had relatively more crystalline regions with inhomogeneous crystal growth, which made it less ductile compared to the PHA/5% FD-TMCNCs. It seems the addition of FD-TMCNC suppressed the growth of inhomogeneous large spherulites and contributed to the increase in elasticity of the composite, promoting fast crystallization. According to the literature, such fast crystallization of the polymer results in a smaller average size of spherulite, and composites with such small spherulites show better mechanical performances than larger spherulite-containing composites [51,52,53]. Thus, in this study, we assumed that 5% of FD-TMCNCs plays a similar role as nucleating agents in PHA. To confirm the effect of TMCNCs on the size distribution of spherulites and the mechanical properties of the composites, further studies can be carried out with visualization analysis using polarized optical microscopy.

## 4. Conclusions

In this work, we demonstrated a method for greatly improving the ductility of PHA composites by blending with surface modified CNC-based fillers. Depending on the silanization and drying process, the morphology of the CNC-based fillers varied, as well as their dispersibility in the PHA composites. Double-silanized CNCs, treated with TEOS and MTMS, and denoted as TMCNCs, allowed the simple preparation of hydrophobically treated fillers, which had good compatibility with the PHA composite. Although the TMCNCs could be prepared by thermal drying, the freeze-dried counterpart had a greatly improved compatibility. The improved compatibility of the TMCNCs was attributed to the (1) hydrophobic surface characteristics of the TMCNCs, and (2) the small and narrow particle size distribution, compared to the MCNCs and TCNCs. The PHA composite containing 5% of freeze-dried TMCNCs showed an increase in elongation at break (up to 300%), with little decrease in Young’s modulus, compared to pure PHA. Therefore, double silanized CNCs can be considered a promising material for improving the processability of PHA, by seemingly working as a nucleating agent. This surface modification strategy with CNC and its compounding in PHA via melt-extrusion show great potential for the designing of a novel grade of biodegradable plastics, meeting industrial demands.

Ultimately, further studies can be conducted on, not only the upstream processing of PHA production, but also the modification of mechanical properties using various materials simultaneously, to acquire a PHA with desirable properties and processability, and available for commercial applications.

## Figures and Tables

**Table 1:** ANOVA table of mechanical properties.

Mechanical Properties	Source of Variation	Sum of Squares	Degree of Freedomdf	Mean Square	F-Value	p-Value
Elongation at break (%)	Regression	64,586.92	3	21,528.97	5.14	0.03
	Residual	33,529.33	8	4191.17		
	Total	98,116.25	11			
Young’s modulus (MPa)	Regression	56.41	3	18.8	46.56	0
	Residual	3.23	8	0.4		
	Total	59.64	11			
Tensile strength at break (MPa)	Regression	185.35	3	61.78	6.237	0.02
	Residual	79.25	8	9.9		
	Total	264.6	11			
